# Medical Opinion and Movement

**Published:** 1908-11-21

**Authors:** 


### Medical Opinion and Movement.

HEISER reports in the last number of the New York *Medical Record* the preliminary notes upon a case of leprosy apparently cured by $x$-ray treatment. The patient was admitted to the San Lazaro Leper Hospital, Manila, on August 12, 1906, with commencing tubercular leprosy. There was infiltration of the alæ nasi, with a tubercle above the left eyebrow and several non-anæsthetic maculæ. Lepra bacilli were obtained from the tubercle and in scrapings from the nasal septum. Treatment was commenced on November 5, 1906, the lesions being exposed "every three days to $x$-rays for ten minutes at a distance of 25 centimetres from the tube. The intensity of the light was just sufficient to give a distinct outline of the bones of the hand." A Grundlach tube and a 45 cm. spark induction coil with a mercury turbine interrupter were used. Gradually the light was applied closer to the lesions. In June 1907 "the affected parts presented almost a normal appearance." In January 1908 "the case was apparently cured from a clinical standpoint; the infiltrations had entirely disappeared, there were no maculæ, and the cosmetic effect was practically perfect so far as the leprosy was concerned." Leprosy bacilli were still present, however, in scrapings from the nasal mucosa. The case is an interesting one, but although the report concludes by stating that "from June 15, 1908, to August 1, 1908, the date of the last observation, it has been impossible to find lepra bacilli in specimens taken from any portion of the patient's body," one would like an equally favourable report some months hence before the case can be accepted as definitely cured. The use of $x$-rays has, we believe, been frequently advocated in the treatment of leprosy, but this appears to be the first case in which the application of the rays has been followed by conspicuous success.

---

FOR several days previous to the operation known as transvesical prostatectomy, Carlier recommends the administration of chloride of calcium in 3- or 4-gram doses, to minimise the risk of post-operative hæmorrhage. The drug should be given apart from milk, since this latter interferes with the action of the salt. Urotropine must be administered with caution, owing to its tendency to provoke hæmorrhage. Before operation, the bladder should be distended with air in preference to fluid. A large abdominal incision should be made so as to bring the field of operation into full view. The incision of the vesical mucous membrance in the neighbourhood of the prostate should be made right round the neck of the bladder, care being taken to leave no tag of membrane jutting out, which might interfere later with the passage of a catheter. Enucleation is effected by splitting the gland with the finger at its anterior commissure, in such a manner as to remove this in two pieces, held together by the posterior commissure. The author recommends section of the vasa deferentia near the root of the scrotum to obviate post-operative orchitis. The prostatic cavity is then irrigated with very warm serum and emptied of clots. A large drainage-tube is next inserted, but the cavity is not plugged. Four irrigations are performed daily through the tube, which is replaced after four days by a smaller one. This latter is left out altogether in five or six days' time, the irrigations being then continued through the wound. After the tenth day the bladder is washed out per urethram.

---

TRAUMATIC rupture of the liver is not only one of the gravest possible sources of internal hæmorrhage, but also one which the medical attendant is particularly at a loss to deal with. On the one hand a small rupture not infrequently, if no other viscus be damaged, heals without interference; on the other an extensive rent is practically always fatal, and direct suture after laparotomy is both unsuccessful and a source of additional danger by disturbing clots that have formed. Mr. J. H. Pringle has attempted to deal with several cases of extensive laceration of the liver which have been under his care in the Glasgow Royal Infirmary, and though he has not yet scored a success, he has by the experience thus gained and by experiments on animals been led to formulate the principles upon which such attempts should be based. He points out that the blood pressure in the portal vein is normally very low, and that when extensive hæmorrhage has occurred from a hepatic rupture the abdominal muscles are often firmly contracted. The intraperitoneal pressure is thus raised to a point which stops the bleeding, and the effused blood then clots.

---

IF now a laparotomy is done, the immediate effect is to reduce the tension in this blood, and allow active hæmorrhage to recommence; yet if nothing be done, and the patient's strength hold out, the effused blood is certain to become infected from the bowel, and death then takes place from peritonitis. So he proposes that as soon as the abdomen is opened the portal vein be compressed digitally at the foramen of Winslow. This has been proved, by experiments on rabbits, to stop all bleeding from the cut surface of the liver, and it proved effective in two patients on whom it was tried after the animal experimentation. He then passes deep sutures into the liver tissue and closes all the rent to which access is possible, dividing the coronary and right lateral ligaments if necessary to reach a posterior rupture. Even then it may not be possible to get at the whole tear, which must in that case be tightly packed. In his last case Mr. Pringle had to deal with an extensive posterior rupture of the liver and with rupture of the right kidney. As much of the former as possible was treated by ligature, and the rest packed. This was successful in stopping hæmorrhage, but the patient died on the fourth day of gangrene of the lung.

As the result of a painstaking investigation, Mr. H. E. Jones formulates in the *London Hospital Gazette* various important conclusions concerning the treatment of Colles' fracture. Full use of skiagraphy was made to discover the exact condition of the bones both before and after reduction, and cases in which this for any reason was not performed are rejected from consideration. It is concluded that in the great majority of cases manipulation does not bring about permanent, if indeed temporary, reduction of the fragments; but such failure of coaptation is of no consequence functionally. In a few cases of very marked displacement permanent improvement in the position of the fragments is secured by forcible extension, with or without anæsthesia. The actual bony deformity is seldom as great as would be supposed from inspection of the wrist after the fracture has occurred. Some degree of impaction is the rule rather than the exception. It is held inadvisable to use any splint in an uncomplicated case if the patient is over 50. Early free movements of the fingers, followed within a week of the accident by passive movements of the wrist and massage are the most important means for securing good results. A sling and bandage are sufficient to secure the necessary rest for the first few days before this treatment is begun. In those who are younger it is not even advisable to attempt reduction unless displacement is excessive, nor to immobilise the limb. In badly comminuted fractures splints are required for a week or ten days, but movements and massage are just as much indicated as in uncomplicated cases. Finally, though splints are unnecessary and good results are achieved slightly more quickly without them, there is no objection to a well-padded Carr's splint, provided the rest of the treatment is carefully attended to; but Gordon's, the pistol, and all other splints which in any way hamper the movements of the fingers are inadmissible.

---

The advantages of analgesic and anæsthetic drugs in the first stage of labour were discussed lately at a meeting of the South Australian branch of the British Medical Association, and found several enthusiastic advocates. Dr. Hamilton, of Adelaide, pins his faith to the combination of morphia and atropine given hypodermically at the beginning of labour. The effects claimed are rapid dilatation of the os and progress of labour without any counterbalancing disadvantages. The same subject, the great value of sedatives and analgesics in the early stages of labour before choloroform is admissible, was considered in greater detail by Dr. Hone, of Semaphore. Chloral he recommends especially for cases of rigid os associated with strong and frequent pains in primiparæ, and Playfair's plan of four fifteen-grain doses every twenty minutes is that which has given him the best results. The application of 10 per cent. cocaine to both surfaces of the os has been advocated, originally by Farrar, for this condition. Dr. Hone has tried it, and thinks it of some use in certain cases, but has given it up in favour of other measures because of the risk of sepsis. Like Dr. Hamilton, he is a firm believer in morphia, of which he used to give a quarter of a grain hypodermically at the commencement of labour. He now combines with this dose $\frac{1}{100}$ grain of hyoscine, and for this treatment he claims most valuable results. The patients generally soon go to sleep, uterine contraction and dilatation of the os proceed rapidly, and the second stage is reached before the patient wakes. A very little chloroform, or even none at all, is then alone required to complete a painless labour in a normal case. Spinal anæsthesia was apparently not discussed at the meeting.

---

Cancer of the mouth and tongue is the subject of an exhaustive paper by Dr. J. C. Warren, of Boston, in the *Annals of Surgery*. In a series of 112 consecutive cases treated by operation in the Massachusetts General Hospital between 1890 and 1904, the percentage free from recurrence after three years is 14.2. The operative mortality varies from 5 per cent. for intra-buccal (Whitehead's) removal of the tongue, upwards to 30 per cent. for operations involving division or resection of the lower jaw. It is very rightly pointed out that the latter proceeding is done for much more advanced cases than the former, and this explains also the larger percentage of cures following the less severe operation. Death is almost always due to local or glandular recurrence, metastases elsewhere being very uncommon. The ideal treatment is held to include preliminary treatment of the buccal cavity, with a view to diminishing its microbic content, and protection of the respiratory canal by drugs (atropine) and by intubation, laryngotomy, or position. The anæsthetic recommended is ether, though how the patient is kept under it does not appear.

---

As regards the actual operation, the removal of the primary lesion with a margin of one inch of healthy tissue is to be aimed at; subject to this it is not necessary always to remove the whole tongue. "Block dissection" of the neck is also always advisable, but in the present state of operative technique is better deferred for a fortnight after the original operation, as a much lower operative mortality is thus secured. This procedure, as devised by Crile, means removal of practically everything in the anterior triangle of the neck except the common carotid artery. The sternomastoid, digastric, and other smaller muscles are sacrificed, without much subsequent inconvenience, it is said. The vagus, phrenic, and hypoglossal nerves on one side can all be dispensed with, on the same authority. Both sides are to be subjected to this drastic treatment if necessary, though caution is necessary before removal of the second internal jugular to make sure that anastomotic channels exist, and the nerves may only be sacrificed on one side. The progress of surgery has proved that to decry sweeping innovations, because they are innovations, is injudicious; but statistics of the mortality and results of this operation will be required before it is at all widely adopted.

THE current number of the *International Journal of Surgery* contains two suggestions in the surgical treatment of minor ailments, which seem worthy of attention. Dr. Stowe advocates the local application of Epsom salts as a primary dressing of burns and scalds. In the case of a burned hand or foot, the limb may be immersed in a concentrated solution of the salts and should be kept there as long as there is pain on removing it from the solution, or the dry salts may be applied to the burn with a wet cloth. It is claimed for this application that it relieves pain almost instantly, inflammation is much reduced and there are no toxic effects. Moreover, it is pointed out that it is a dressing which generally can be quickly procured. The second suggestion consists in a method of operation for ingrowing toe-nail. Dr. Noble maintains that the usual method of avulsion is most unsatisfactory and usually leads to recurrence. He produces local anæsthesia by cocaine injections, carrying the needle always towards the end of the toe. With a straight bistoury he transfixes the overlapping flesh, beginning the incision at the base of the nail. Keeping the knife on the nail the incision is carried to the end of the toe. The flap is laid back and all the diseased flesh dissected from the skin. Ulcerations are scraped off. The nail is then lifted and its under side scraped clean but not cut. A pledget of cotton wool is placed under the nail to raise it slightly and the flap placed back. A dressing is applied and bandaged tightly to prevent hæmorrhage. As Dr. Noble claims to have great success with this method of treatment, and to have cured himself after many years of torture, it is worthy of consideration.

"MICROSPHYGMY" forms the subject of an interesting article in *Le Progrès Médical*, by Drs. Bournville, Richet and Saint-Girons. The condition was first described by M. Variot in 1898, and he gave the name to a syndrome characterised by a triad of symptoms, microsphygmy, idiotcy, and ichthyosis. Three types of "microsphygmy," or small pulse, may be distinguished, in which the pulse is either feeble, but perceptible, appearing as a fine undulation; or is altogether imperceptible (asphygmy); or is only perceptible occasionally and in certain conditions. According to these authors the condition extends to all the peripheral arterial trunks, but is most easily detected in the brachial. It is not due to an arterial lesion, but rather a physiological defect in the arterial system, possibly a spasm of the muscular tunics dependent upon some change in the sympathetic system. Variations in the degree of microsphygmy under certain conditions—external temperature, inhalation of amyl-nitrite, etc.—are characteristic of the condition. It is found in females only and associated with some condition of idiocy; on the other hand Variot's third symptom of ichthyosis is not always present. Other dystrophies and malformations are frequently associated with it. Family history generally shows a tuberculous, syphilitic, or alcoholic taint. Such rare and extreme conditions are chiefly interesting in suggesting the cause for similar phenomena of a milder type in comparatively normal individuals.

PROFESSOR O. Laurent, writing in the *Revue Medico-Sociale*, advocates surgical intervention in certain arthritic conditions of a gouty nature. Operation of any kind is to be avoided during an acute attack of gout. It must be borne in mind that operations are liable to provoke an attack in those so disposed. But apart from these considerations the author finds that no little useful surgical assistance can be afforded to the gouty by the removal of tophi and by the curetting of joints that have developed chronic deposits and thickenings. He cites a case of a man, aged 50, with the gouty diathesis, but never having suffered a typical acute attack of gout, who had developed considerable swelling and deformity of some of the small joints of the left foot. The condition was especially marked at all the articulations of the large toe and at the metatarsophalangeal joint of the second toe, and at this point there was a sinus leading down to the joint and to the first two phalanges. On pressure numerous tophi could be felt around the joints. A plantar incision was made, large masses of tophi were removed with the curette, and the affected joints were curetted and the first two phalanges resected. The operation was followed by primary union, and in a short time the foot resumed its normal proportions, and the patient was able to use it freely. Professor Laurent thinks such operative procedures are indicated in cases of a gouty origin in which there is chronic inflammation of long standing with ulceration or persistent pain, provided the general condition is good, and in certain cases of extreme deformity he advocates the amputation of one or more of the toes.

---

THE "intradermo-reaction" is yet another form of local reaction to tuberculin which was introduced by M. Mantoux at a recent meeting of the Académie de Médecine. A drop of solution containing one-hundredth of a milligramme of tuberculin is injected into the skin. A positive reaction is shown by a white or pink infiltration, often surrounded by a halo of erythema. The height of the reaction is attained at the end of 48 hours, and after that it gradually subsides. The author considers it is preferable to the cuti-reaction, and, above all, to the ophthalmo-reaction, as it is free from the dangers attaching to the latter. According to M. Mantoux, positive reactions are rare in infants of the first year, but increase in frequency with the advance in years. In youth they may occur five times in six. They are supposed due to latent tuberculosis. In the case of a suspicious lesion, therefore, it must not be concluded from a positive reaction that the lesion is definitely tuberculous, as the reaction may be due to some hidden focus, such as a mediastinal gland. On the other hand, if the reaction be negative the presence of tuberculous disease may almost with certainty be rejected. The ophthalmo-reaction promised great things in its early days, but the publication of several cases in which its use has been fraught with disastrous results has naturally made many chary of resort to it. A safe and equally definite result such as the cuti-reaction, and possibly also this intradermo-reaction, appear to offer is welcome.

